# The NMR Structure of Human Obestatin in Membrane-Like Environments: Insights into the Structure-Bioactivity Relationship of Obestatin

**DOI:** 10.1371/journal.pone.0045434

**Published:** 2012-10-04

**Authors:** Begoña O. Alén, Lidia Nieto, Uxía Gurriarán-Rodríguez, Carlos S. Mosteiro, Juan C. Álvarez-Pérez, María Otero-Alén, Jesús P. Camiña, Rosalía Gallego, Tomás García-Caballero, Manuel Martín-Pastor, Felipe F. Casanueva, Jesús Jiménez-Barbero, Yolanda Pazos

**Affiliations:** 1 Área de Endocrinología Molecular y Celular, Instituto de Investigación Sanitaria (IDIS), Complejo Hospitalario Universitario de Santiago de Compostela (CHUS), SERGAS, Santiago de Compostela, Spain; 2 CIBER Fisiopatología de la Obesidad y Nutrición (CB06/03), Instituto de Salud Carlos III, Santiago de Compostela, Spain; 3 Universidad de Santiago de Compostela, Santiago de Compostela, Spain; 4 Instituto de Investigación Sanitaria (IDIS), Complejo Hospitalario Universitario de Santiago de Compostela (CHUS), Santiago de Compostela, Spain; 5 Centro de Investigaciones Biológicas, CIB-CSIC, Madrid, Spain; 6 Unidad de Resonancia Magnética, RIAIDT, Universidad de Santiago de Compostela, Campus Sur, Santiago de Compostela, Spain; Consiglio Nazionale delle Ricerche, Italy

## Abstract

The quest for therapeutic applications of obestatin involves, as a first step, the determination of its 3D solution structure and the relationship between this structure and the biological activity of obestatin. On this basis, we have employed a combination of circular dichroism (CD), nuclear magnetic resonance (NMR) spectroscopy, and modeling techniques to determine the solution structure of human obestatin (**1**). Other analogues, including human non-amidated obestatin (**2**) and the fragment peptides (6–23)-obestatin (**3**), (11–23)-obestatin (**4**), and (16–23)-obestatin (**5**) have also been scrutinized. These studies have been performed in a micellar environment to mimic the cell membrane (sodium dodecyl sulfate, SDS). Furthermore, structural-activity relationship studies have been performed by assessing the *in vitro* proliferative capabilities of these peptides in the human retinal pigmented epithelial cell line ARPE-19 (ERK1/2 and Akt phosphorylation, Ki67 expression, and cellular proliferation). Our findings emphasize the importance of both the primary structure (composition and size) and particular segments of the obestatin molecule that posses significant α-helical characteristics. Additionally, details of a species-specific role for obestatin have also been hypothesized by comparing human and mouse obestatins (**1** and **6**, respectively) at both the structural and bioactivity levels.

## Introduction

Obestatin, a 23-amino acid peptide derived from the ghrelin peptide precursor (preproghrelin), was identified in 2005 as a physiological opponent of ghrelin [1]. Obestatin was originally isolated from the stomach and has subsequently been shown to be a circulating peptide whose secretion is pulsatile. Obestatin displays an ultradian rhythmicity similar to that of ghrelin and GH secretion [2]. Moreover, obestatin was shown to bind selectively to the orphan receptor GPR39, which belongs to the same family as the ghrelin receptor GHS-R1a and the motilin receptor [2], [3].

Although obestatin has been known as a controversial ghrelin-associated peptide due to the lack of reproducible biological actions on feeding, additional activities for this molecule have been reported [4]. In particular, the proliferative abilities of obestatin were first demonstrated in human retinal pigmented epithelial cells [5], and the intracellular mechanisms responsible of this action were later elucidated in human gastric cancer cell lines [6], [7], thus defining the functionality to this biologically active peptide.

The ERK1/2 molecules control a large number of different or even opposed cellular processes such as proliferation, survival, development, stress response, and apoptosis [8]. Akt is a serine/threonine kinase that acts as central player in the regulation of several cell functions: protein synthesis, cell survival (one of the main factors in many cancer types) and metabolism (glucose uptake, lipid homeostasis and protein synthesis) [9]. Deregulation of these signals usually provokes diseases, such as cancer or diabetes. Once obestatin activates GPR39, two routes are triggered in parallel: i) sequential activation of Gi, PI3K, novel PKCε and Src and the subsequent ERK1/2 activation; and ii) a β-arrestin 1-mediated signaling pathway that involves the recruitment of Src to the β-arrestin 1 scaffolding complex, thus causing Akt phosphorylation. In this way, Src acts as a switch that activates matrix metalloproteinases (MMPs) to initiate the proteolytic release of the EGF-like ligands onto the cell surface, which later bind to EGFR. The binding of the ligands leads to receptor dimerization, which activates the intrinsic kinase and the specific binding sites of phosphorylation, including PI3K. This kinase activation allows for Akt phosphorylation in the A-loop (T308) and the HM (S473) by PDK1 and mTORC2, respectively. The activated Akt then inactivates the TSC1/TSC2 heterodimer to activate mTORC1 and phosphorylate downstream targets, including p70S6K1 [7]. The fact that obestatin modulates cell proliferation, especially that of gastric cancer cells, suggests the involvement of this peptide in diverse processes, such as the repair of gastric mucosal damage or as a fuel for gastric cancer cell proliferation. Subsequently, obestatin displayed a novel role as an autocrine/paracrine regulator of adipogenesis and adipocyte metabolism [10], [11]. All of the effects observed for obestatin indicate that this molecule is a biologically relevant peptide and not only a non-functional connective peptide.

**Figure 1:**
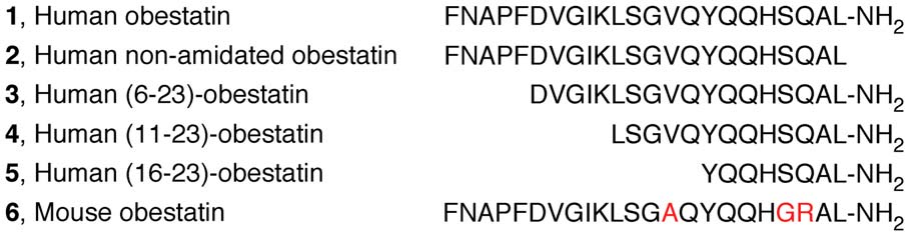
Primary structure of the obestatins used in this work: human obestatin (1), human non-amidated obestatin (2), human (6–23)-obestatin (3), human (11–23)-obestatin (4), human (16–23)-obestatin (5) and mouse obestatin (6). The red-labeled residues in **6** are different from those of **1**.

Regarding the structure of obestatin, this peptide contains 23 amino acids with a post-translational amide modification on the C-terminus. It is believed that this modification is essential for the bioactivity of obestatin [1]. Recently, the relationship between the primary structure and the bioactivity of mouse obestatin has been studied, and it was concluded that the N-terminal 13 residues of obestatin exhibit structural behavior most similar to that of the full peptide [12]. Additionally, CD and NMR studies have been performed to determine the possible secondary structure of mouse obestatin (**6**) and its (11–23)-obestatin truncated isoform in the presence of DPC/SDS micelles. The results showed that both peptides assume regular secondary structures at the C-terminus and that carboxy-amidation is a prerequisite for the biological activity because it is necessary to induce and to stabilize the regular conformations [13]. More recently, Subasinghage et al. have performed a structure-bioactivity study using human obestatin. These authors described the effects of human obestatin and its (11–23)-obestatin isoform in rats [14]. In addition to these studies, the availability of new data regarding the signaling mechanisms of obestatin in human cell lines [5]–[7] has prompted us to develop further studies on this topic. In this context, we have used CD, NMR spectroscopy, and modeling techniques to determine the solution structure of human obestatin (**1**), human non-amidated obestatin (**2**) and the fragment peptides (6–23)-obestatin (**3**), (11–23)-obestatin (**4**), and (16–23)-obestatin (**5**) in SDS micelles (Figure 1). Furthermore, comparisons have been made between the structural characteristics and the biological activities observed for these peptides, as determined by their capabilities to stimulate Akt and ERK1/2, the expression of the proliferation marker Ki67 and the cellular proliferative capabilities [15] in the human retinal pigmented epithelial cell line ARPE-19. Additionally, the details of the species-specific role of obestatin have also been deduced by comparing human and mouse obestatin (**1** and **6**, respectively).

**Figure 2:**
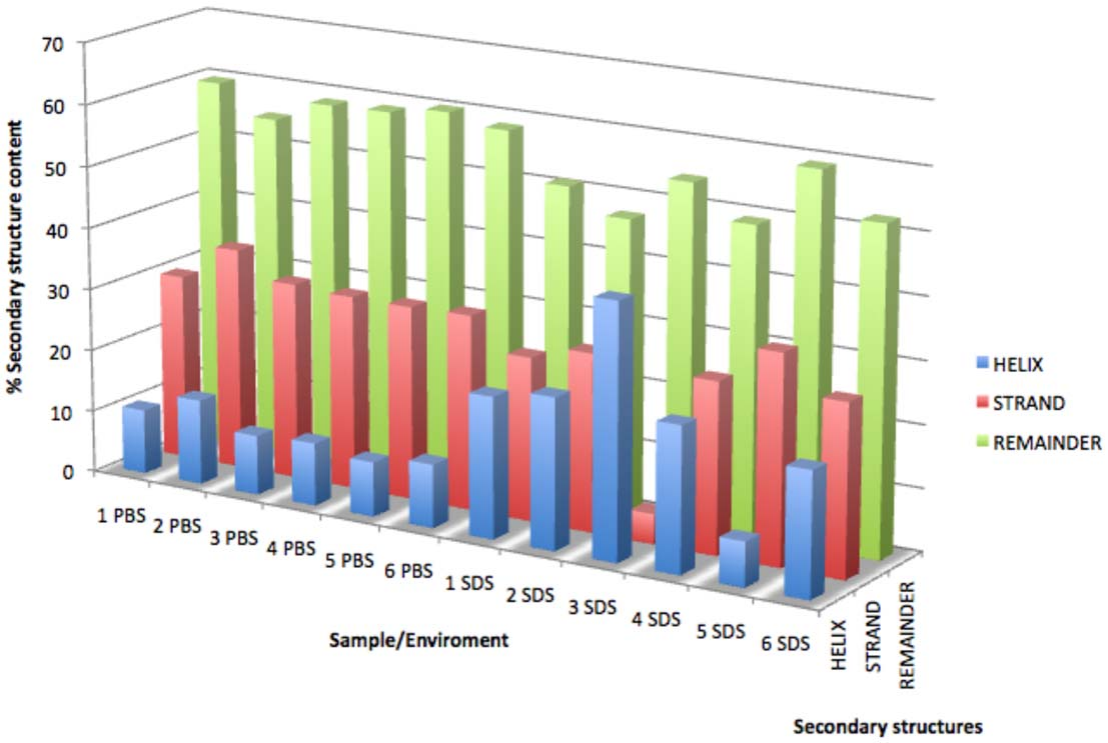
Secondary structure analyses performed using the DICHROWEB web server with the CONTINLL algorithm and reference data set 4. The relative amounts of α-helix and β-sheet were determined by adding together the contributions from helix 1 plus helix 2 and strand 1 plus strand 2, respectively, whereas the amounts of β-turn and random structure were read directly from the output. The peptides clearly showed greater helicity in SDS than in PBS.

## Materials and Methods

### Materials

Human obestatin (**1**) and mouse obestatin (**6**) were obtained from California Peptide Research Inc. (Napa, CA, US). Human non-amidated obestatin and human (16–23)-obestatin were purchased from Bachem Ltd. (St. Helens, UK). Human (6–23)-obestatin was obtained from Biomedal (Sevilla, ES). Human (11–23)-obestatin was obtained from Peptides International (Louisville, KY, US).

**Figure 3:**
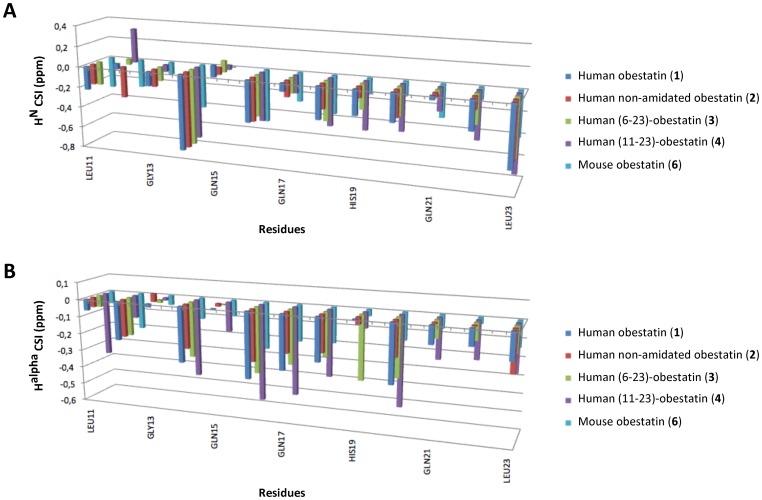
Plot of the chemical shifts indices (CSIs) for residues at the C-termini of the studied peptides. (A) CSIs of the backbone amide protons (H^N^) and (B) CSIs of the alpha protons (H^alpha^). The peptide sequence (**6**) is aligned with that of the other peptides. The CSI is defined as δ^obs^ – δ^random-coil^.

**Figure 4:**
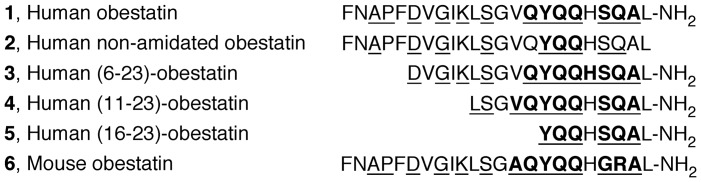
Secondary helical structure based on the H^N^ and H^alpha^ chemical shifts indices determined using the RCI server. The underlined residues were predicted to have secondary helical structure based on the H^N^ and H^alpha^ chemical shifts indices that were determined using the RCI server (http://wishart.biology.ualberta.ca/rci). The residues within helical structures that extended over more than two residues are represented in bold; these residues are located primarily at the C-terminus.

The rabbit polyclonal IgG antibodies against phospho-p44/42 mitogen-activated protein kinase (MAPK), p44/42 MAPK, phospho-Akt HM(S473), and Akt HM(S473) were purchased from Cell Signaling Technology (Beverly, MA, US). The rabbit polyclonal IgG antibodies against GPR39 and actin were obtained from Abcam (Cambridge, UK). The anti-rabbit IgG horseradish peroxidase was purchased from Jackson ImmunoResearch Europe Ltd. (Suffolk, UK).

**Figure 5:**
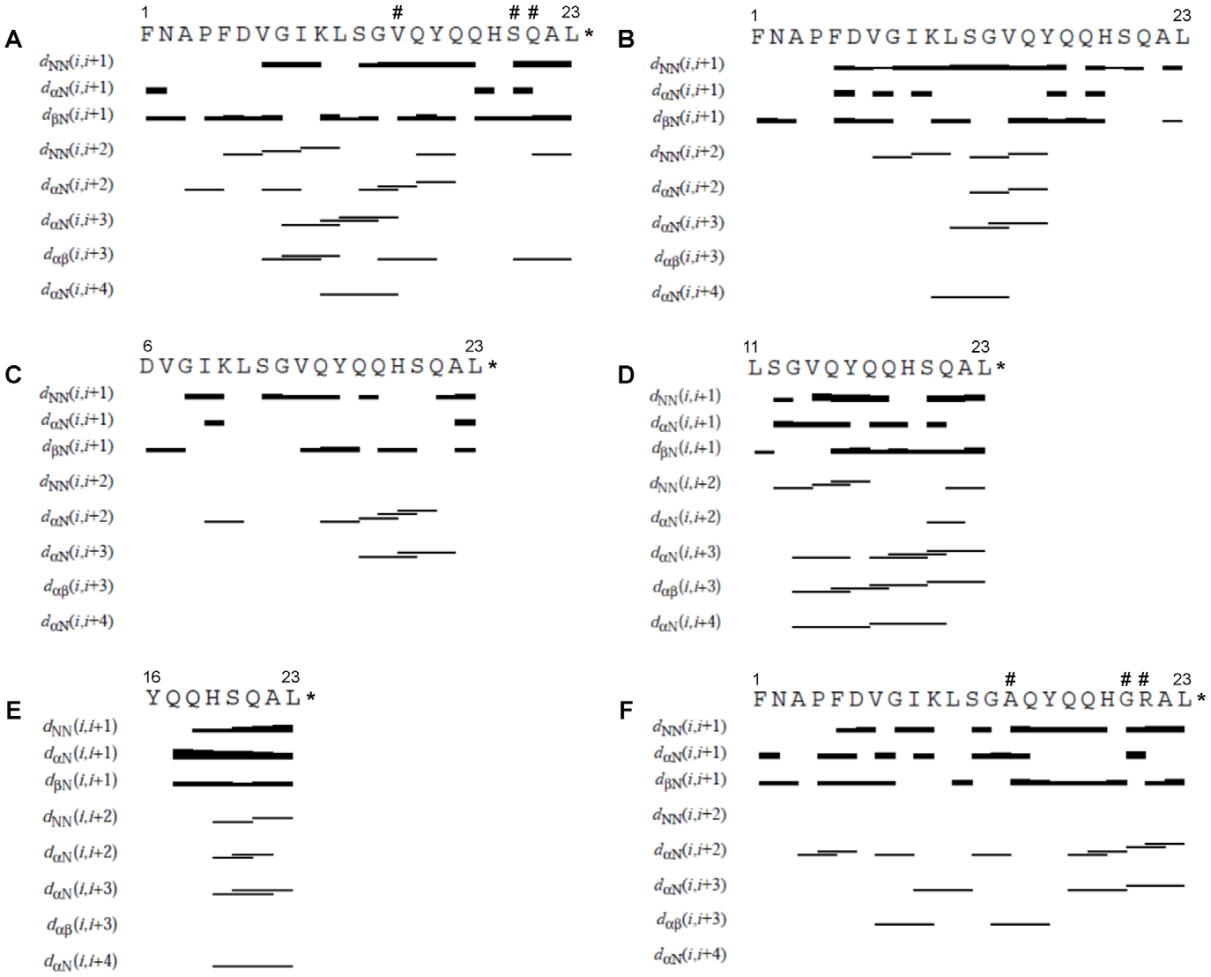
Summary of sequential and medium-range NOE connectivities involving the NH, Hα and Hβ protons of the peptides in SDS micelles, as derived from CYANA calculation. The thickness of the bar indicates the intensities of the NOEs for the following peptides: (A) human obestatin (**1**), (B) human non-amidated obestatin (**2**), (C) human (6–23)-obestatin (**3**), (D) human (11–23)-obestatin (**4**), (E) human (16–23)-obestatin (**5**) and (F) mouse obestatin (**6**). The asterisk (*) represents the C-terminal amidation of the molecule. The dagger (#) represents the differences between human obestatin (**1**) and mouse obestatin (**6**).

### Cell culture

The retinal pigmented epithelium cell line ARPE-19 was cultured as described by the supplier (ATCC, Manassas, VA, US). Briefly, ARPE-19 cells were seeded in 100-mm dishes and cultured in DMEM:F12 medium supplemented with 10% (v/v) fetal bovine serum (FBS), 100 U/mL penicillin G, 100 mg/mL streptomycin sulfate and 2.5 mM L-glutamine with 5% CO_2_ at 37°C.

**Table 1:** Structural statistics for the ensemble of the best 20 structures of human obestatin (1), its fragments and mouse obestatin (6).

	Human obestatin (1)	Human non amidated-obestatin (2)	Human (6–23)-obestatin (3)	Human (11–23)-obestatin (4)	Human (16–23)-obestatin (5)	Mouse obestatin (6)
**Experimental restraints** [a]						
Sequential distances	267	145	145	184	123	284
Medium-range distances (i–j) <5	99	28	31	63	38	68
Long-range distances (i–j) ≥5	2	0	0	0	2	7
Total	368	173	176	247	163	359
Final CYANA target function value (Å^2^) [b]	0.18	0.22	0.02	0.11	0.27	0.04
**RMS deviations from ideal geometry** [c]						
Bond lengths (Å)	0.005	0.005	0.004	0.004	0.004	0.005
Bond angles (°)	0.7	0.7	0.6	0.5	0.5	0.6
**RMSD to mean coordinates (Å)** [d]	(14–20)	(14–20)	(14–20)	(14–20)	(16–23)	(14–20)
Backbone N, C^α^, C'	0.42±0.21	1.49±0.56	0.55±0.23	0.09±0.04	0.49±0.24	0.46±0.39
All heavy atoms	1.19±0.38	2.69±0.78	1.24±0.27	0.49±0.22	1.50±0.40	0.93±0.52
**Ramachandran plot statistics** [c]						
Most favorable regions (%)	71.4	72.3	69.6	70.5	60.0	77.4
Additional allowed regions (%)	26.4	27.2	30.4	29.5	38.7	22.6
Generously allowed regions (%)	0.0	0.1	0.0	0.0	1.3	0.0
Disallowed regions (%)	2.2	0.3	0.0	0.0	0.0	0.0

[a] The final CYANA target function value was computed for the structures calculated using CYANA.

[b] Average values of the 20 final energy-minimized CYANA conformers.

[c] Calculated using PROCHECK-NMR.

[d] Atomic differences are given as the average RMS difference of the mean coordinate structure (mean).

### Immunoblotting analysis

Serum-starved cells were stimulated with the peptides for the indicated time period and doses at 37°C. The medium was then aspirated, and the cells were lysed in ice-cold lysis buffer [RIPA buffer: 50 mM Tris-HCl (pH 7.2), 150 mM NaCl, 1 mM EDTA, 1% (v/v) NP-40, 0.25% (w/v) Na-deoxycholate, protease inhibitor cocktail (1∶100, Sigma Chemical Co., St. Louis, MO, US), phosphatase inhibitor cocktail (1∶100, Sigma Chemical Co., St. Louis, MO, US)]. The soluble cell lysates were pre-cleared by centrifuging at 13,000×g for 15 min. The protein concentration was evaluated using the QuantiProTM BCA Assay kit (Sigma Chemical Co., St. Louis, MO, US). The same amount of protein for each sample was separated on 10% sodium dodecyl sulfate (SDS)/polyacrylamide gels and transferred to nitrocellulose membranes (Bio-Rad, Hercules, CA, US). The blots were incubated with 5% non-fat milk in a Tris buffer solution containing Tween-20 (TBST) [20 mM Tris-HCl (pH 8.0), 150 mM NaCl, 0.1% (v/v) Tween-20, used for all incubation and washing steps] for 1 h. The blots were then incubated for 1 h with the corresponding antibodies, according to the manufacturer's instructions, and were subsequently incubated with the peroxidase-conjugated IgG antibody. After washing, the signals were visualized using an ECL plus Western Blotting Detection System (GE-Amersham, Buckinghamshire, UK). The blots shown are representative of three experiments. The image processing was performed using the NIH Image Software ImageJ 1.38x.

**Figure 6:**
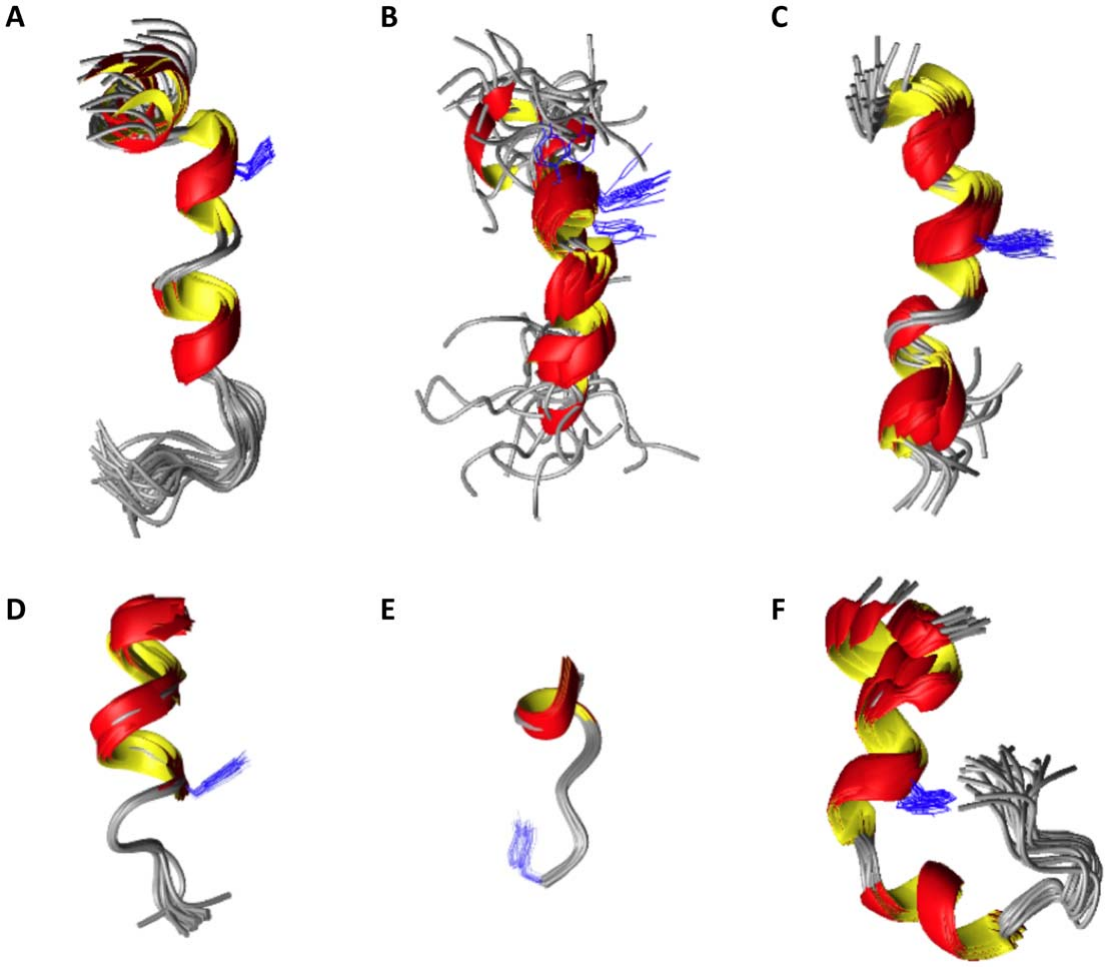
Superimposition of the 20 best representative structures of peptides 1 to 6, as calculated from the NMR data for the peptides in SDS micelles. (A) Human obestatin (**1**), (B) human non-amidated obestatin (**2**), (C) human (6–23)-obestatin (**3**), (D) human (11–23)-obestatin (**4**), (E) human (16–23)-obestatin (**5**) and (F) mouse obestatin (**6**). The Tyr16 side chain is shown in blue.

**Figure 7:**
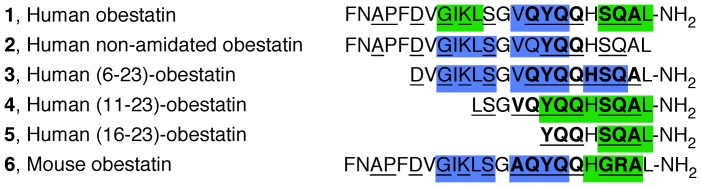
Comparison between the secondary helical structure based on the H^N^ and H^alpha^ chemical shifts indices determined using the RCI server and the secondary helical structure obtained in our structures. The underlined residues were predicted to have secondary helical structure based on the H^N^ and H^alpha^ chemical shifts indices obtained using the RCI server (http://wishart.biology.ualberta.ca/rci). The residues within helical structures that extended over more than two residues are represented in bold; these residues are located primarily at the C-terminus. The secondary helical structure obtained in our structures, as calculated by CYANA, included α-helix formation (green labels) and 3_10_-helix formation (blue labels).

### Small Interfering RNA (siRNA) silencing of gene expression

The following double-stranded siRNA duplexes of GPR39 were used (Thermo Fisher Scientific, Dharmacon, Lafayette, CO, US; ON-TARGETplus SMART pool L-005569-00-0005, Human GPR39, NM_001508): 3′-UCCAAUAUGUCCAUCUGUA-5′, 3′-GCGCGAAACCAGCCAAUUC-5′, 3′-GAGGCUGAUUGUUGUGACA-5′, and 3′-AACCAGAUUCGGAGGAUCA-5′. A non-silencing RNA duplex was used as a control for all siRNA experiments. The ARPE cells were transfected using Lipofectamine 2000 (Invitrogen; CA, US).

**Figure 8:**
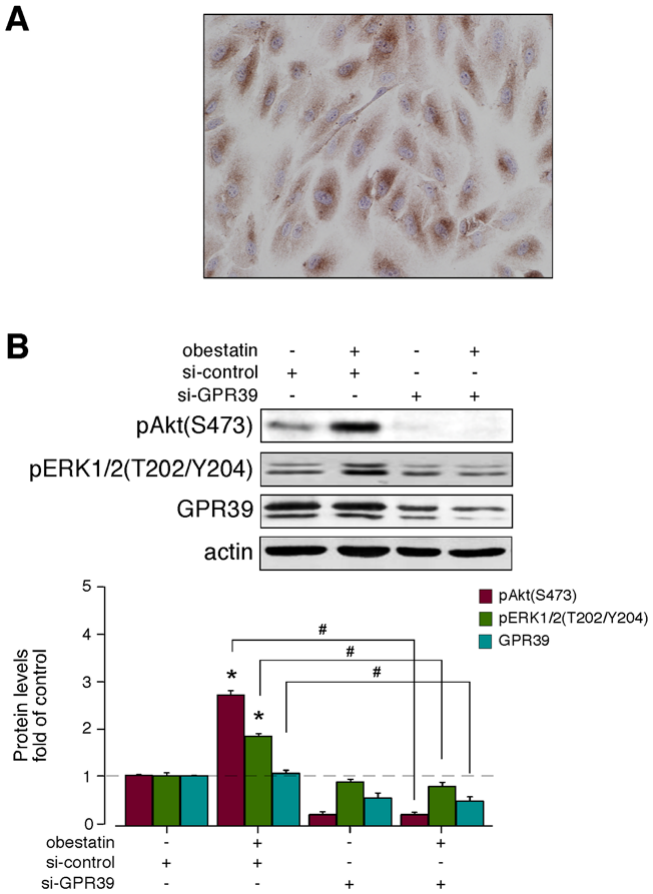
Analysis of the expression and functionality of GPR39 in ARPE-19 cells. (A) Immunocytochemical detection of GPR39 in ARPE-19 cells (objective magnification of 20x). (B) The effect of siRNA depletion of GPR39 on pAkt(S473) and pERK1/2(T202/Y204) in ARPE-19 cells after human obestatin treatment (**1**, 100 nM, 10 min). The ARPE-19 cells were transfected with GPR39 siRNA prior to obestatin **1** treatment. Equal amounts of protein in each sample were used to assess the expression of GPR39 by western blotting. The GPR39 level was expressed as the fold change relative to the control siRNA-transfected cells (mean ± SE). The protein expression was normalized relative to actin. The data are expressed as the mean ± SE. The asterisk (*) denotes *P*<0.05 when comparing the treated control siRNA group with the control siRNA group; the dagger (#) denotes *P*<0.05 when comparing the GPR39 siRNA group with the control siRNA group.

### Immunocytochemistry detection of Ki67

The ARPE-19 cells were cultured at a density of 4×10^3^ cells per well in the culture medium described above on 8-well Lab-Tek II chamber slides covered with CC2 glass slide coverslips. After 2 days, the medium was renewed, and the cells were cultured in a serum-free medium (300 μL) for 24 h. The cells were then treated with FBS (10% v/v), human obestatin (**1**; 100 and 200 nM), human non-amidated obestatin (**2**; 100 and 200 nM) and the fragment peptides (6–23)-obestatin (**3**; 100 and 200 nM), (11–23)-obestatin (**4**; 100 and 200 nM), (16–23)-obestatin (**5**; 100 and 200 nM) and mouse obestatin (**6**; 100 and 200 nM) in fresh DMEM:F12. After 24 h, the intact cells were fixed in 96% ethanol for 1 h. The immunocytochemical technique was automatically performed using an AutostainerLink 48 instrument (Dako, Glostrup, DK). The FLEX primary antibody to Ki-67 (clone MIB 1; Dako) was used. En Vision FLEX/HRP (Dako) was employed as a detection system. Briefly, the procedure comprised the following steps: 1) epitope retrieval in 10 mM citrate buffer (pH 6.0) using a microwave (750 W, 10 min); 2) incubation with peroxidase-blocking agent (5 min); 3) incubation with primary antibody (30 min); 4) incubation with labeled polymer-horseradish peroxidase (HRP, dextran polymer conjugated with HRP and affinity-isolated goat anti-mouse immunoglobulins; 30 min); 5) incubation with a diaminobenzidine (DAB) chromogen substrate solution (10 min); and 6) counterstaining with Harris hematoxylin (9 min). In all cases, triplicate dishes were used for each experimental point.

**Figure 9:**
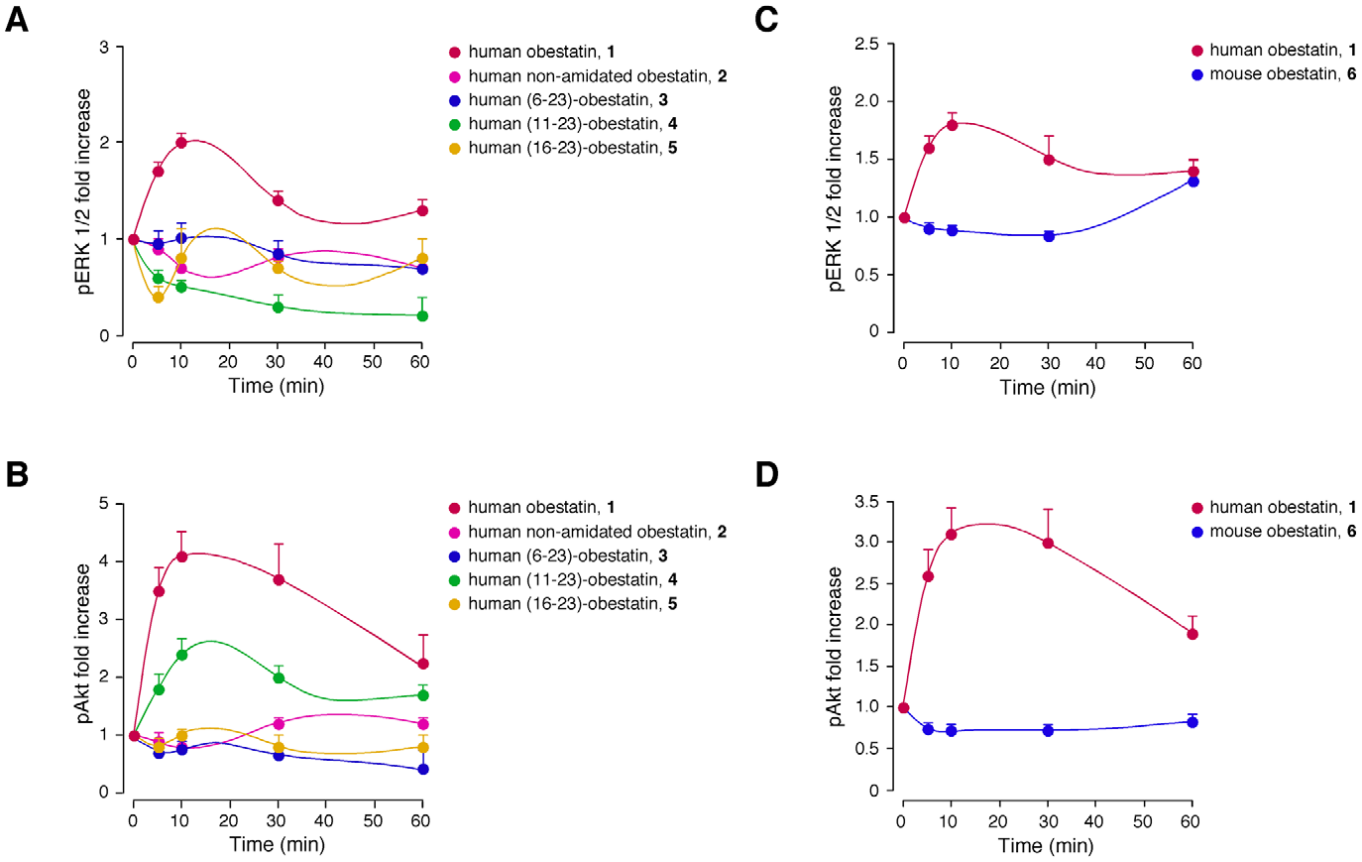
Time-course of the effect of the different peptides on ERK1/2 [pERK 1/2 (T202/Y204)] and Akt [pAkt HM (S473)] phosphorylation in ARPE-19 cells. The serum-starved cells were treated with the peptides (200 nM) at 37°C for the indicated times. The cells were lysed and analyzed using SDS-PAGE with specific antibodies. ERK1/2 and Akt phosphorylation were quantified using densitometry and expressed as the fold change relative to the phosphorylation obtained for unstimulated cells (mean ± SE of five independent experiments).

### Cell proliferation assay

The cell proliferation was measured using a BrdU cell proliferation enzyme-linked immunosorbent assay (ELISA) kit (Roche Diagnostics, Mannheim, DE). The BrdU assay was performed according to the manufacturer's protocol. ARPE-19 cells were cultured in a 96-well multiplate at a density of 2×10^3^ cells per well in the culture medium described above for 24 h. The procedure comprised the following steps: 1) 0% FBS for 24 h; 2) stimulation with FBS (10% v/v), human obestatin (**1**, 100 nM), human non-amidated obestatin (**2**, 100 nM) and the fragment peptides (6–23)-obestatin (**3**, 100 nM), (11–23)-obestatin (**4**, 100 nM), (16–23)-obestatin (**5**, 100 nM) and mouse obestatin (**6**, 100 nM) in serum-deprived medium for 48 h; 3) incubation with BrdU-labeling solution (10 μL, 3 h, 37°C); 4) removal of the labeling solution and fixing with FixDenat solution (200 μL, 30 min, 25°C); 5) incubation with an anti-BrdU-peroxidase (POD) antibody solution (100 μL, 90 min, 25°C); and 6) washing followed by the addition of the substrate solution (100 μL, 30 min). The BrdU incorporation was quantified using the spectrophotometric absorbance (370 nm) measured with a Reader VersaMaxPLUS. The mean absorbance of the control cells represented 100% cell proliferation, and the mean absorbance of the treated cells was related to the control values to determine the sensitivity. In all cases, each experimental point was replicated eight times.

**Figure 10:**
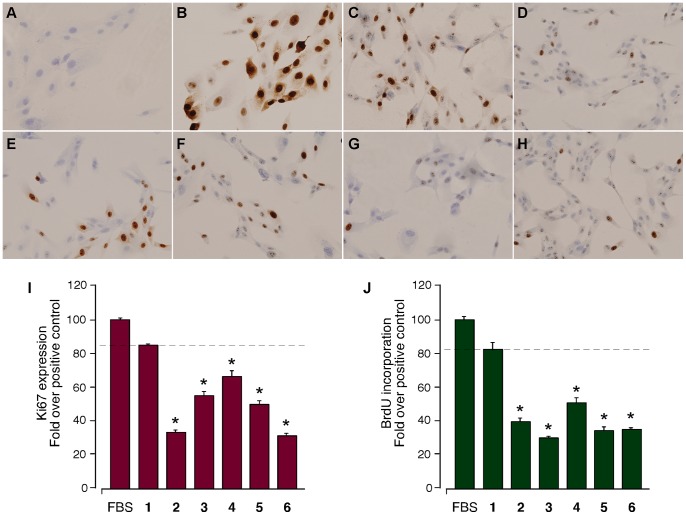
Immunocytochemical analysis of the Ki67 expression and BrdU incorporation in ARPE-19 cells. Immunocytochemical analysis of the Ki67 expression in ARPE-19 cells after 24 h of proliferation. A) Control. B) 10% FBS (v/v). C) 100 nM human obestatin (**1**). D) 100 nM human non-amidated obestatin (**2**). E) 100 nM human (6–23)-obestatin (**3**). F) 100 nM human (11–23)-obestatin (**4**). G) 100 nM human (16–23)-obestatin (**5**). H) 100 nM mouse obestatin (**6**). The magnification was 20x. I) Quantification of the immunocytochemical expression of Ki67 in ARPE-19 cells after treatment with 10% FBS (v/v; 100±2), 100 nM human obestatin (**1**; 84±1%), 100 nM human non-amidated obestatin (**2**; 33±2%), 100 nM human (6–23)-obestatin (**3**; 56±3%), 100 nM human (11–23)-obestatin (**4**; 67±3%), 100 nM human (16–23)-obestatin (**5**; 50±2%) and 100 nM mouse obestatin (**6**; 32±2%). The expression of Ki67 was expressed as the fold change relative to the expression level in FBS-treated cells in the positive control (mean ± SE). J) BrdU incorporation in ARPE-19 cells after treatment with 10% FBS (v/v; 100±1), 100 nM human obestatin (**1**; 84±4%), 100 nM human non-amidated obestatin (**2**; 39±2%), 100 nM human (6–23)-obestatin (**3**; 30±1%), 100 nM human (11–23)-obestatin (**4**; 52±3%), 100 nM human (16–23)-obestatin (**5**; 34±3%) and 100 nM mouse obestatin (**6**; 35±1%). The BrdU incorporation was expressed as the fold change relative to the level in FBS-treated cells in the positive control (mean ± SE). The data are expressed as the mean ± SE. The asterisk (*) denotes *P*<0.05 when comparing the peptide-treated ARPE-19 cells groups with the human obestatin (**1**)-treated group.

### Immunocytochemistry detection of GPR39

The ARPE-19 cell samples were processed using standard procedures [10]. The slides were consecutively incubated with 1) anti-GPR39 rabbit polyclonal antibody (1∶500) in Dako ChemMate antibody diluent (Dako, Glostrup, DK); 2) EnVision™ peroxidase rabbit (Dako, Carpinteria, CA, US) as the detection system; and 3) 3,3′-diaminobenzidine-tetrahydrochloride (Dako Liquid DAB + Substrate-chromogen system). The cell and tissue sections were faintly counterstained with Harris hematoxylin.

### NMR spectroscopy

Samples for the NMR experiments were prepared by dissolving peptides in 500 μL of a d25-SDS aqueous solution (9∶1 H_2_O:D_2_O, PBS buffer at pH 6.5) to make a final concentration of approximately 0.8–1.0 mM and a peptide/SDS ratio of approximately 1/70. The NMR spectra were recorded at 298 K using Bruker 600 and 700 MHz spectrometers equipped with a triple-resonance Z gradient probe and processed using XWIN-NMR software (Bruker Inc.; Billerica, MA, US). The resonance of 2,2,3,3-tetradeutero-3-trimethylsilylpropionic acid (TSP) was used as a chemical shift reference in the ^1^H NMR experiments (δ TSP = 0 ppm). The one-dimensional (1D) ^1^H homonuclear spectra were recorded in the Fourier mode using quadrature detection. The two-dimensional (2D) ^1^H homonuclear TOCSY (total correlation spectroscopy) and NOESY (nuclear Overhauser effect spectroscopy) spectra were collected in the phase-sensitive mode using time-proportional phase increments in t1. For each of these experiments, 512 t1 increments were used. The free induction decay in t2 consisted of 2048 data points over a spectral width of 6009.615 Hz. In general, 4096×1024 data points were collected for each block, and 96 transients were collected for the 2D experiments. The TOCSY spectra were recorded using the MLEV-17 pulse sequence with mixing times (spin-lock) of 60–80 ms. The NOESY experiments were acquired with mixing times of 250–400 ms. The experimental data were acquired and processed using the TopSpin™ (Bruker Inc; Billerica, MA, US) program on a PC station. The data matrices were multiplied by a qsine function in both dimensions and then zero-filled to 1024 data points in F1 prior to the Fourier transformation. The peptide resonance assignments were obtained using standard strategies based on the 2D NMR experiments.

### Structure calculation

The peak lists for the NOESY spectra recorded with a 0.25 s mixing time were generated by interactive peak picking using the CARA software [16]. The NOESY cross-peak volumes were determined using the automated peak integration routine implemented in CARA. Conversion of NOE peak intensities to distance restraints was done using automatic calibration as implemented in CYANA 2.1 [17]. The three-dimensional (3D) structures of human obestatin (**1**) and its derivatives were determined using the standard protocol of combined automated NOE (nuclear Overhauser effect) assignment and the structure calculation implemented in the CYANA 2.1 program. Seven cycles of combined automated NOESY assignment and structure calculations were followed by a final structure calculation. The structure calculation started in each cycle from 100 randomized conformers, and the standard simulated annealing schedule was used. The 20 conformers with the lowest final CYANA target function values were retained for analysis and passed to the next cycle. Weak restraints on the phi/psi torsion-angle pairs and on the side-chain torsion angles between tetrahedral carbon atoms were applied temporarily during the high-temperature and cooling phases of the simulated annealing schedule to favor the permitted regions of the Ramachandran plot and the staggered rotamer positions, respectively. The list of upper-distance bonds for the final structural calculation consists of unambiguously assigned upper-distance bonds and does not require the possible swapping of diastereotopic pairs. The 20 conformers with the lowest final CYANA target function values were subjected to restrained energy-minimization in a water shell using the AMBER 8.0 program [18]. The resulting 20 energy-minimized CYANA conformers represent the solution structures of human obestatin (**1**) and its derivatives. The MOLMOL program was used to visualize the 3D structures [19]. CYANA was used to obtain statistics on the target function values, restraint violations and Ramachandran plots according to the PROCHECK-NMR conventions [20]. The RMSD (root mean square deviation) values were calculated using CYANA for the superpositions of the backbone N, Cα and C' atoms and the heavy atoms throughout the protein. To obtain the RMSD value of a structure represented by a bundle of conformers, all of the conformers were superimposed on the average structure, and the average of the RMSD values between the individual conformers and their average coordinates was calculated.

### CD spectroscopy

CD experiments of the peptides were performed using a 720-Jasco spectropolarimeter (Tokyo, JP) and a 1-mm-path-length quartz cuvette. The CD spectra of the peptides in the SDS micellar solution were recorded using a H_2_O solution containing 40 μM peptide and SDS micelle at a concentration of 2.8 mM (25°C). In all cases, 25 mM NaH_2_PO_4_/Na_2_HPO_4_ buffer was used to maintain the pH at 6.5. The CD spectra presented are the average of 5 accumulations from 190 to 250 nm, which were recorded with a bandwidth of 1 nm and a scanning speed of 20 nm/min. During all of the measurements, the trace of the high-tension voltage remained less than 700 V, which should ensure the reliability of the obtained data [21]. Baselines of either solvent or micellar solutions without peptide were subtracted from each respective sample to yield the contribution of the sample. The secondary structure composition was estimated using the DICHROWEB web server [22], [23] with the following algorithms: CONTINLL, involving 2 different reference data sets, and K2d [23].

### Data analysis

All of the data are reported as the mean ± SE. A statistical ANOVA analysis was performed using an analysis of variance with the Bonferroni post hoc test. Values of *P*<0.05 were considered to be statistically significant and are marked with an asterisk (*).

## Results and Discussion

### 1. Circular dichroism

Circular dichroism is a suitable and rapid approach to provide information about the secondary structural features of peptides such as obestatin in solution [24]. First, the influence of SDS micelles on the secondary structure of the different peptides was studied. A preliminary scanning of the conformational preferences of the peptides was performed in 25 mM PBS and in an SDS micelle solution. SDS was adopted based on the works of Schwyzer, who hypothesized that previous contact with the membrane is essential for the peptide to adopt the proper conformation to further interact with its receptor [25].

As shown in Figure S1, the CD spectra of the peptides (40 μM) suggested that random coil conformations were prevalent in PBS, whereas the spectra recorded in the micelle solution indicated the presence of a certain amount of α-helical structure (Figure S2). Both human (**1**) and mouse obestatin (**6**) exhibited similar tendencies (Figure S3).

The secondary structure analyses were performed using the DICHROWEB web server [22], [23] with the following algorithms: CONTINLL, which incorporated 2 different reference data sets, and K2d [23]. The conformational weights of the secondary structures obtained with CONTINLL were paired with 2 different data sets for the data ranging between 190 and 240 nm. Although an important caveat of this method is that the reference data sets are primarily appropriated for aqueous environments, it is nonetheless evident that the peptides in SDS have the greatest helicity (Table S1). An example of the obtained results is represented in Figure 2.

### 2. Structure determination using nuclear magnetic resonance (NMR)

The NMR spectra of the peptides were collected in the presence of the SDS micelles. Peptides **1–6** provided well-dispersed 2D spectra. A small number of cross-peaks were observed for peptides **2** and **3**. Figure S4 and Figure S5 exemplify the quality of the spectra, of which the amide region of the 2D NOESY spectra of human obestatin (**1**) and its truncated analogue (**4**) are shown. The assignment process was straightforward because most of the proton resonances of these peptides at 298 K and pH 6.5 were well resolved and narrow. The chemical shift assignment and collection of the NOE data were performed by analyzing the 2D TOCSY and NOESY spectra using the CARA software and following standard procedures [16]. The proton chemical shifts of the peptides in SDS micelles are summarized in the Supporting Information: human obestatin (**1**), Table S2; human non-amidated obestatin (**2**), Table S3; human (6–23)-obestatin (**3**), Table S4; human (11–23)-obestatin (**4**), Table S5; human (16–23)-obestatin (**5**), Table S6; and mouse obestatin (**6**), Table S7.

The experimental NMR data were used to generate 3D models of peptides **1–6**. The three-dimensional structures were calculated using the CYANA software based on the inter-proton distance restraints (sequential and medium-range NOE-derived restraints). The best 20 structures, out of 50 calculated, were chosen according to the lowest values of the penalty (f) for the target function. A subsequent energy refinement was performed in explicit solvent using the AMBER program. The corresponding results, including the further analysis of the resulting structures performed using MOLMOL and PROCHECK-NMR, are shown below.

#### 2. 1. Backbone HN amide and H-alpha ^1^H chemical shifts

First, the variation of the backbone amide H^N^ and H^alpha^ chemical shift values was monitored for significant changes that might have structural relevance. These chemical shifts in peptides **1–6** were, on average, shifted upfield in relation to the typical values observed for random coils, as might be expected for helical structures (Figure 3). Furthermore, the chemical shift analysis performed using the RCI server [26] predicted helical conformations for the residues located mainly at the C-termini of the peptides (Figure 4).

Figure 3 shows the chemical shift indices (CSI) for the H^N^ and H^alpha^ protons of the residues at the C-termini of some of these peptides. The CSIs for the H^N^ and H^alpha^ protons of human obestatin (**1**) between residues Tyr16 and Leu23 are generally (with a few exceptions) negative and much stronger than those of the non-amidated obestatin (**2**), indicating that the latter has a smaller percentage of helical structure at the C-terminus. The CSIs for the H^N^ and H^alpha^ protons of human (6–23)-obestatin (**3**) generally indicate a higher percentage of helical structure relative to (**1**), suggesting that the lack of the first 5 amino acids does not have an important conformational impact on the structure.

The truncated peptide **4**, which lacks the first 10 residues, has the strongest negative CSIs for both H^N^ and H^alpha^ protons, thus providing further support for the formation of an α-helix at the C-terminus of this peptide. In the case of mouse obestatin (**6**), the negative CSIs for H^N^ and H^alpha^ are indicative of an α-helix, although the comparison with the human obestatin (**1**) shows considerable variations along the sequence without a clear trend.

#### 2. 2. NOE pattern

Figure 5 contains a summary of all (short, medium and long range) connectivities deduced from an analysis of the NOESY spectra of the peptides. It has been previously reported that poorly ordered structures of obestatin were observed in water solutions. These peptides generally exhibited bend structures in the central Lys10-Ala14 fragment [13], [14]. For peptides **1–6** in buffered SDS micelles, the backbone and side-chain proton NMR resonances could be completely assigned in a sequential manner with the aid of the TOCSY and NOESY spectra. A significant population of conformations containing an “ordered” α-helix is present, with characteristic short-range dNN(i, i+1) and medium-range dαN(i, i+3) and dαβ (i, i+3) NOE connectivities [16]. Fairly strong dNN(i, i+1) and dαN(i, i+1) NOEs were observed for most of the residues, although some gaps resulted from resonance overlap.

The data that were obtained clearly suggest the presence of a significant population of ordered α-helical structures for these peptides in SDS micelle solutions. In fact, for human obestatin (**1**), clear medium- and long-range connectivities were observed including 1) medium-range Hαi/HNi+3 (Gly8/Leu11, Lys10/Gly13, Leu11/Val14) and Hαi/Hβi+3 (Val7/Lys10, Gly8/Leu11, Gly13/Tyr16, Ser20/Leu23) connectivities and 2) a long-range Hαi/HNi+4 (Lys10/Val14) connectivity. For the non-amidated peptide **2**, fewer medium-range connectivities were observed relative to the amidated peptide **1**, including 1) medium-range Hαi/HNi+3 (Leu11/Val14, Gly13/Tyr16) connectivities but no Hαi/Hβi+3 connectivities and 2) a long-range Hαi/HNi+4 (Lys10/Val14) connectivity. For the truncated human (6–23)-obestatin (**3**), fewer medium-range connectivities were observed: 1) medium-range Hαi/HNi+3 (Gln17/Ser20, His19/Ala22) connectivities but no Hαi/Hβi+3 connectivities were observed, and 2) no long-range Hαi/HNi+4 connectivities were observed. The presence of medium-range connectivities (Hαi/HNi+3, Hαi/Hβi+3) and long-range connectivities (Hαi/HNi+4) from Gly13 to Leu23 confirmed the presence of a fairly well defined α-helical structure at the C-terminus of human (11–23)-obestatin (**4**): 1) medium-range Hαi/HNi+3 (Gly13/Tyr16, Gln17/Ser20, Gln18/Gln21, Ser20/Leu23) and Hαi/Hβi+3 (Gly13/Tyr16, Gln15/Gln18, Gln17/Ser20, Ser20/Leu23) connectivities were observed, and 2) long-range Hαi/HNi+4 (Gly13/Gln17, Glu17/Gln21) connectivities were observed. The observed structure of the human (16–23)-obestatin (**5**) is not as well defined as that of **4**. Only residues His19/Leu23 are involved in α-helix formation: 1) medium-range Hαi/HNi+3 (His19/Ala22, Ser20/Leu23) connectivities and no Hαi/Hβi+3 connectivities were observed, and 2) a long-range Hαi/HNi+4 (His19/Leu23) connectivity was also observed. A fairly similar pattern of NOEs, especially at the C-terminal region of the peptide, was found in the SDS micelles compared with the structure previously reported in DPC/SDS micelles [13] for mouse obestatin (**6**): 1) medium-range Hαi/HNi+3 (Ile9, Ser12, Gln17/**Gly20**, **Gly20**/Leu23) and Hαi/Hβi+3 Val7/Lys10, Gly13/Tyr16) connectivities were observed, and 2) no long-range Hαi/HNi+4 connectivities, however, were observed (bold residues are different from those of **1**).

#### 2. 3. 3D structures

The best 20 structures, which possessed the lowest total energies, were considered as representatives of human obestatin (**1**) and its analogues. None of these structures had NOE violations greater than 0.2 Å or dihedral angle violations greater than 2°. A summary of the structural characteristics of the different peptide ensembles is given in Table 1. The PROCHECK analysis of the 20 structures showed that almost all of the residues are in the most favored and ‘additionally allowed’ regions of the Ramachandran plot. For instance, for **1**, less than 3% of the residues were found outside the sterically allowed region of the Ramachandran plot. This fact is mostly due to Ala3, which is situated in the less-defined region of the structure (Table 1).

A view of the structures obtained for **1–6** superimposed along their backbones is shown in Figure 6. These representations show that these peptides primarily adopt an α-helical conformation at the C-terminus (Figure 7).

The analysis of the bundle of 20 structures obtained for human obestatin (**1**) demonstrated that in most of the structures, residues Gly8-Leu11 were involved in an α-helix, whereas residues Val14-Gln17 were involved in a 3_10_ helix. In a few structures, residues Ser20-Leu23 were further involved in an α-helix. This helical pattern induced under our SDS micelles conditions appears to be fairly similar to that previously reported in 33% TFE-water for the same peptide [14]. Mean atomic RMSD values for the backbone atoms (5–15) of 0.52±0.25 Å and for the heavy atoms (5–15) of 1.09±0.32 Å were calculated for the best 20 conformers of **1**, and these values are fairly similar to those reported by Subasinghage under those experimental conditions [14]. For the human non-amidated obestatin (**2**), the formation of a 3_10_ helix between residues Gly8-Ser12 and Val14-Gln17 was observed. Regarding peptide **3**, a 3_10_ helix between residues Gly8-Ser12, Val14-Gln17 and His19-Gln21 was found. For the human (11–23)-obestatin (**4**), the α-helix structure extended from Tyr16 to Leu23. The small peptide **5** displays a short α-helix from Ser20 to Leu23. Finally, our data suggest that the mouse obestatin (**6**) contained a 3_10_ helix between residues Gly8-Ser12 and Ala14-Gln17 and an α-helix between His19-Ala22. The calculated 3D structures also corroborate some details that were previously obtained from the qualitative ^1^H chemical shift analysis described above. As can be observed from these data, the amide moiety in **1** appears to be important for stabilizing the α-helix conformation between residues Ser20 and Leu23, and this moiety is absent in the non-amidated peptide (Figure 6A and 6B).

### 3. Proliferation

#### 3. 1. GPR39 siRNA depletion

GPR39 simultaneously signals via two parallel pathways, using either heterotrimeric G proteins or multifunctional adapters such as β-arrestin 1. Although recent work has identified this receptor as a target for obestatin action [10], [11], no data are available for the proposed cell model of ARPE-19 cells, a human retinal pigment epithelial cell line.

Therefore, the endogenous expression of GPR39 was first analyzed using immunocytochemistry (Figure 8A). Next, the effect of acute GPR39 deficiency was determined using siRNA. Under these conditions, the constructs exhibited a decrease in GPR39 expression by 55±1% (Figure 8B). In the presence of a non-targeting control siRNA, the phosphorylation of human obestatin-activated Akt(S473) and ERK1/2(T202/Y204) were similar to that observed without any transfection. The silencing of GPR39 subsequently decreased the levels of pAkt(S473) and pERK1/2(T202/Y204) with respect to the siRNA control (to 94±3% and 60±5%, respectively) following treatment with human obestatin (**1**, 100 nM) for 10 min (Figure 8B).

#### 3. 2. ERK1/2 and Akt activation

We examined the proliferative capabilities of the peptides by investigating ERK1/2 and Akt activation [pERK1/2(T202/Y204) and pAkt(S473)]. The curves obtained after the treatment of ARPE-19 cells with the peptides (100, 200 and 500 nM) exhibited a dose-dependent pattern with a maximum at 200 nM (data not shown). This maximum is the concentration reflected in Figure 9. The pattern of ERK1/2 phosphorylation (Figure 9A) reached maximal levels within 10 min of human obestatin stimulation (**1**, 200 nM) and decreased to basal levels after 60 min. Conversely, the pAkt(S473) maximal levels were reached after 10 min of stimulation with human obestatin (**1**, 200 nM), and the activation was maintained for at least 60 min (Figure 9B). In contrast, the human non-amidated obestatin (**2**) did not influence pAkt(S473), and exhibit even a slight inhibitory effect on pERK1/2(T202/Y204). From these data, it can be deduced that amidation is a prerequisite for maintaining the bioactivity of obestatin. Obestatins **3** and **5** had no effect on either pAkt(S473) or pERK1/2(T202/Y204). Intriguingly, the obestatin analogue **4** induced selective coupling to the Akt signaling pathway and exhibited no effect on the ERK1/2 route over the time course of the assay. This particular ligand is processed from full-length obestatin and is present in the stomach [1]. In this sense, this peptide appears to selectively enhance a restricted subset of active GPR39 conformations that are capable of promoting specific aspects of the signaling pathway within the GPR39-induced network, particularly β-arrestin-mediated signaling mechanisms. This result suggests that this molecule may act as a potential β-arrestin-biased agonist [27]. To date, biased ligands have been identified for different G protein-coupled receptors, denoting a new class of pharmacological ligands capable of selective modulation [28].

Regarding the species-specific role of obestatin, the effect of human (**1**) and mouse (**6**) obestatins on pERK1/2(T202/Y204) and pAkt(S473) was compared under the same experimental conditions described previously. Obestatin (**6**) treatment did not induce any significant change in either pERK1/2(T202/Y204) or pAkt(S473) during the tested period (Figure 9C and 9D). This result demonstrates that, in this system, the changes in primary (Val14Ala, Ser20Gly and Gln21Arg) and secondary structure between species are critical for the activity. Thus, a species-specific behavior occurs.

#### 3. 3. Ki67 expression and proliferation

The activation of the ERK1/2 and Akt signaling network results in the activation of a series of transcription factors that induce alterations in the expression of a variety of genes involved in the stimulation of cell proliferation. Among these factors, Ki67 is a labile non-histone nuclear protein that is intimately involved in the cell cycle [29]. The expression of Ki67 exhibits a good relationship with the growing fraction in several system models, and this factor is not expressed during the repair processes of DNA [30]. Thus, Ki67 is considered to be a marker of cell proliferation.

Consequently, the mitogenic effect of the peptides was tested by analyzing the expression of Ki67 in ARPE-19 cells using an immunocytochemical analysis. The experiments were performed at 24 h post-stimulation with the different peptides (100 nM). Figure 10 shows a representative experiment of the six independent assays. Human obestatin (**1**) exhibited a clear increase in immunostaining for Ki67 (Figure 10C), which was comparable to the positive control [10% FBS (v/v); Figure 10B]. The negative control (without stimuli) is shown in Figure 10A. In accordance with the results from the immunoblots, the expression of Ki67 was marginally detected for non-amidated obestatin (**2**) and mouse obestatin (**6**) (Figure 10D and 10 H, respectively). Alternatively, human (6–23)-obestatin (**3**) and human (16–23)-obestatin (**5**) exhibited weak Ki67 expression (Figure 10E and 10G, respectively), and the expression was higher for human (11–23)-obestatin (**4**) (Figure 10F). These results (Figure 10I) resemble the proliferative capabilities of human obestatin (**1**; 84±4%) and the different peptides measured in terms of BrdU incorporation, in which only peptide **4** (52±3%) exhibited a slight effect (Figure 10J).

Classically, agonists of the G protein-coupled receptor have been thought to display a linear efficacy in which activation of the receptor-related signaling network (e.g., G protein signaling, receptor phosphorylation, β-arrestin recruitment and internalization) is associated with the degree of the receptor activation, and this activation ranges from partial to full. Nonetheless, it is now recognized that β-arrestins initiate and determine the signals with different spatial and temporal patterns, thus resulting in singular cellular and pathophysiological consequences. This fact introduces a new concept that proposes the efficacy of G protein-coupled receptors as pluridimensional factors. This conclusion means that G protein-coupled receptors exhibit different active conformations that are able to trigger either the full range of receptor-associated activities or a subset of these activities. In this context, it appears that human obestatin (**1**) stabilizes the GPR39 conformations that are necessary for the complete signaling associated with the G protein and β-arrestin downstream pathways. This ligand action is clearly determined by amidation at the C-terminus of human obestatin (**1**), which was shown to be essential for signaling in all downstream pathways of the G protein- and β-arrestin-mediated signaling with the subsequent activation of ERK1/2 and Akt, respectively. As deduced from the structural studies, human non-amidated obestatin (**2**) does not possess the α-helical pattern of **1**, such as the α-helix formed between Ser20 and Leu23 and the α-helix formed from Gly8 to Lys11. Instead, a 3_10_ helix appears between Gly8 and Ser12. This structural change may be responsible for the negligible effect of **2** on the expression of Ki67, which is not comparable to that caused by human obestatin (**1**). To date, Ki67 has not been unequivocally associated with either the G protein- or β-arrestin-mediated signaling. In fact, although Ki67 has been recognized as a relevant prognostic and predictive marker for proliferation, the exact intracellular regulation remains obscure. For **3**, the absence of the first five residues appears to be the key to the inability of this peptide to stabilize the GPR39 conformations associated with the activation of the G protein-dependent and β-arrestin-dependent signaling transductions. Furthermore, the impaired effect of peptide **3** on the expression of Ki67 compared with that induced by **1** reinforces the previous hypothesis. Regarding human (11–23)-obestatin (**4**), the loss of the first ten residues leads to a significant change in structure. This peptide presents the longer segment of an α-helix spanning from Tyr16 to Leu23. The biological data show that this ligand induces a selective coupling to only the β-arrestin portion of the GPR39 downstream signaling pathways. This evidence suggests that this peptide stabilizes a receptor conformation distinct from that induced by **1**. Interestingly, **4** exhibited a higher level of Ki67 expression than the other truncated obestatin analogues did. It is tempting to speculate that the structure of this ligand represents a key element needed for the stabilization of the GPR39 conformation coupled to β-arrestin signaling, thus controlling its interaction patterns and related functions. Finally, human (16–23)-obestatin (**5**) possesses a helical structure at its C-terminus. Nevertheless, its small size does not seem to be able to modulate the full range of GPR39 activities. Altogether, these results support the notion that more than one active conformation of GPR39 indeed exists and that the different ligands are able to stabilize a different subset of the available receptor conformations.

The behavior of mouse obestatin (**6**) is also unique. This peptide is the same size as **1** and is also subject to amidation at its C-terminus. Moreover, **6** displays a characteristic pattern of helix sets: Gly8-Ser12, Ala14-Gln17, and His19-Ala22. Indeed, the first two helical regions are coincident with the 3_10_-helix fragment present in **3**. However, the presence of this type of helix in **6** does not result in the activation of the human GPR39 receptor in ARPE-19 cells. In fact, mouse obestatin (**6**) did not activate either G protein-dependent or β-arrestin-dependent signal transduction over the time period studied. Additionally, this peptide failed to induce Ki67 expression, which is similar to the behavior of human non-amidated obestatin (**2**). An inspection of the primary sequence indicated that the differences in primary structure between **1** and **6** are limited to only three amino acid residues, namely Val14Ala, Ser20Gly and Gln21Arg. The major dissimilarity is observed for Arg21, which can be positively charged. Spatially, this residue is located only two residues away from the neighboring amidated Leu23. Thus, these structural differences may be responsible for the different bioactivities observed. Thus, in our biological model system, the activity of obestatin is species-specific.

## Conclusions

A structure-activity relationship for obestatin has been derived by employing this peptide and several analogues as ligands for the seven-transmembrane receptor GPR39. The analysis of the data suggests that amidation at the C-terminus of human obestatin (**1**) is essential for this molecule to adopt an α-helix structure. This α-helix exists between Ser20 and Leu23 and from Gly8 to Lys11. The presence of this structure correlates with the stabilization of the GPR39 conformations that are necessary for the full range of receptor activities, e.g., G protein-dependent and β-arrestin-dependent signaling. Indeed, the change of this α-helix to a 3_10_ helix or the loss of this α-helical pattern can be correlated with the absence of complete activation, as observed for non-amidated obestatin (**2**). Additionally, GPR39 is able to adopt multiple active conformations, which are related to the activation of specific signaling mechanisms. In particular, human (11–23)-obestatin (**4**) is able to induce selective coupling to a portion of the downstream signaling pathways, e.g., β-arrestin-dependent signaling. This observation supports the idea that **4** stabilizes a receptor conformation different from that induced by human obestatin (**1**). Most likely, this activity is related to the presence of the α-helix segment from Tyr16 to Leu23. Because this peptide is present in the stomach, it might represent the first example of an endogenous biased ligand for GPR39. Finally, mouse (**6**) and human obestatin (**1**) exhibit clear conformational differences beyond their differences in primary structure. The mouse analogue adopts a distinct three-dimensional structure, which cannot activate human GPR39. This evidence supports the existence of a species-specific activity. Overall, the data presented herein provide a new structural background, which could be useful for the development of particular ligands that are able to discernibly improve, diminish or inhibit specific aspects of the GPR39-associated signaling pathways.

## Supporting Information

Figure S1 **Far-UV CD spectra of the peptides in 25** **mM PBS under identical conditions.** The concentration was 40 μM in all samples. Color code: solid blue, human obestatin (**1**); dotted blue, non-amidated obestatin (**2**); solid red, human (6–23)-obestatin (**3**); dotted red, (11–23)-obestatin (**4**); and solid green, (16–23)-obestatin (**5**).
(TIF)

Figure S2 **Far-UV CD spectra of the peptides in 2.8** **mM SDS under identical conditions.** The concentration was 40 μM in all samples. Color code: solid blue, human obestatin (**1**); dotted blue, non-amidated obestatin (**2**); solid red, human (6–23)-obestatin (**3**); dotted red, (11–23)-obestatin (**4**); and solid green, (16–23)-obestatin (**5**).
(TIF)

Figure S3 **Far-UV CD spectra of human obestatin (1) and mouse obestatin (6) in 25** **mM PBS or 2.8** **mM SDS under identical conditions.** The concentration was 40 μM in all samples. Colour code: solid red, human obestatin (**1**) in SDS; dotted red, human obestatin (**1**) in PBS; solid blue, mouse obestatin (**6**) in SDS; and dotted blue, mouse obestatin (**6**) in PBS.
(TIF)

Figure S4 **Amide region of 2D NOESY spectra for human obestatin (1) in SDS micelles.**
(TIF)

Figure S5 **Amide region of 2D NOESY spectra for human (11–23)-obestatin (4) in SDS micelles.**
(TIF)

Table S1 **Quantification of obestatins secondary structure by CD.** The following table describes the quantification of the CD spectra in two different environments. The quantification was performed using the DICHROWEB server and the data were fit using K2d and CONTINLL. The latter was paired with two different data sets for data ranging from 190 to 240 nm. The NRMSD refers to the quality of the curve fitting based on the reference data sets.
(TIF)

Table S2 **^1^H chemical shifts (p.p.m.) for human obestatin (1) in SDS-d_25_ micelles at 298 K.**
(TIF)

Table S3 **^1^H chemical shifts (p.p.m.) for human non-amidated obestatin (2) in SDS-d_25_ micelles at 298 K.**
(TIF)

Table S4 **^1^H chemical shifts (p.p.m.) for human (6–23)-obestatin (3) in SDS-d_25_ micelles at 298 K.**
(TIF)

Table S5 **^1^H chemical shifts (p.p.m.) for human (11–23)-obestatin (4) in SDS-d_25_ micelles at 298 K.**
(TIF)

Table S6 **^1^H chemical shifts (p.p.m.) for human (16–23)-obestatin (5) in SDS-d_25_ micelles at 298 K.**
(TIF)

Table S7 **^1^H chemical shifts (p.p.m.) for mouse obestatin (6) in SDS-d_25_ micelles at 298 K.**
(TIF)
