# Supplementary figures and images for: The NMR Structure of Human Obestatin in Membrane-Like Environments: Insights into the Structure-Bioactivity Relationship of Obestatin
Source: PLoS One. 2012 Oct 4;7(10):e45434. doi: 10.1371/journal.pone.0045434 (PMC3464274; doi:10.1371/journal.pone.0045434)

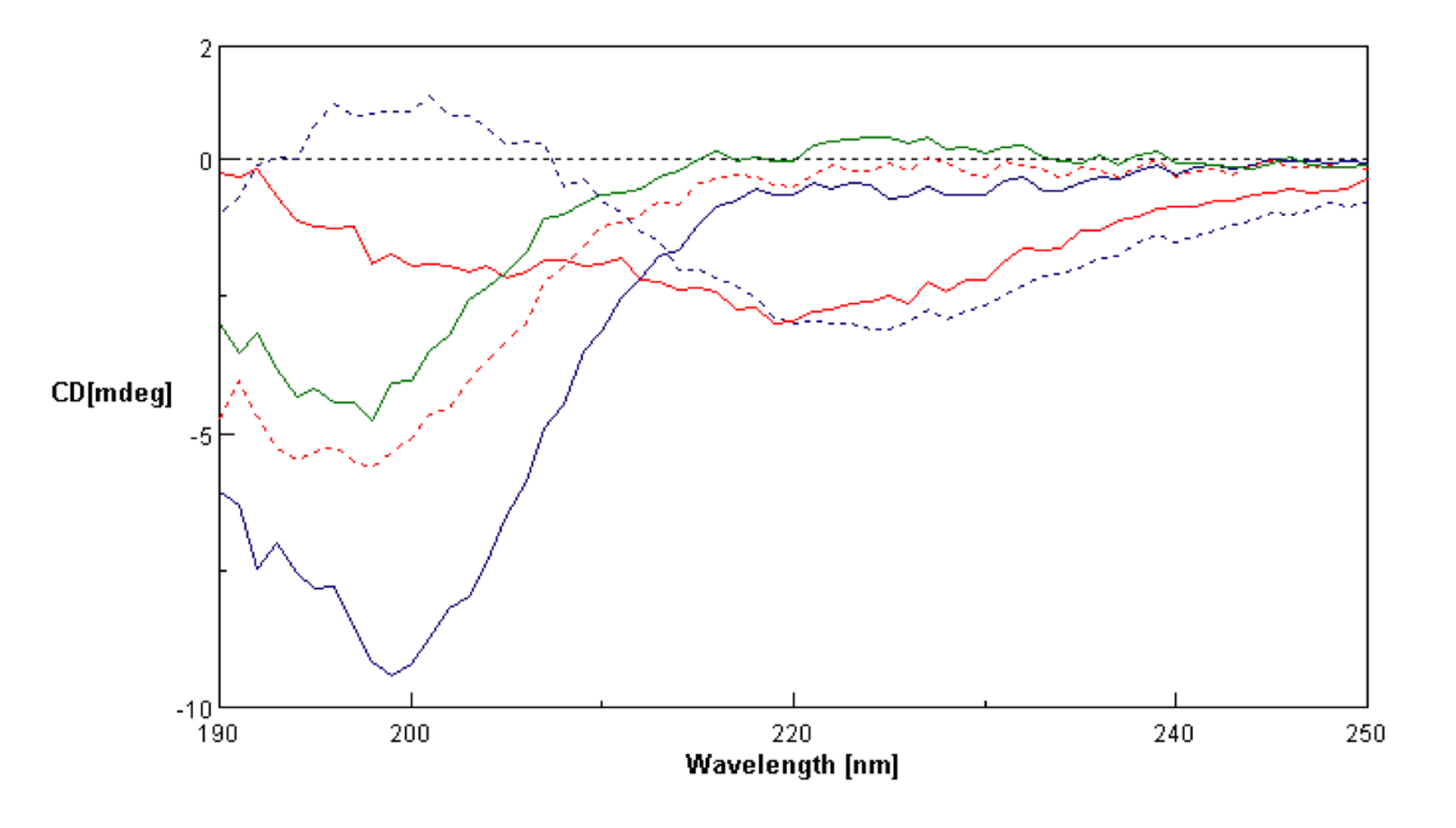

Supplement: Figure S1 — Far-UV CD spectra of the peptides in 25 mM PBS under identical conditions. The concentration was 40 μM in all samples. Color code: solid blue, human obestatin (1); dotted blue, non-amidated obestatin (2); solid red, human (6–23)-obestatin (3); dotted red, (11–23)-obestatin (4); and solid green, (16–23)-obestatin (5). (TIF) [file pone.0045434.s001.tif]

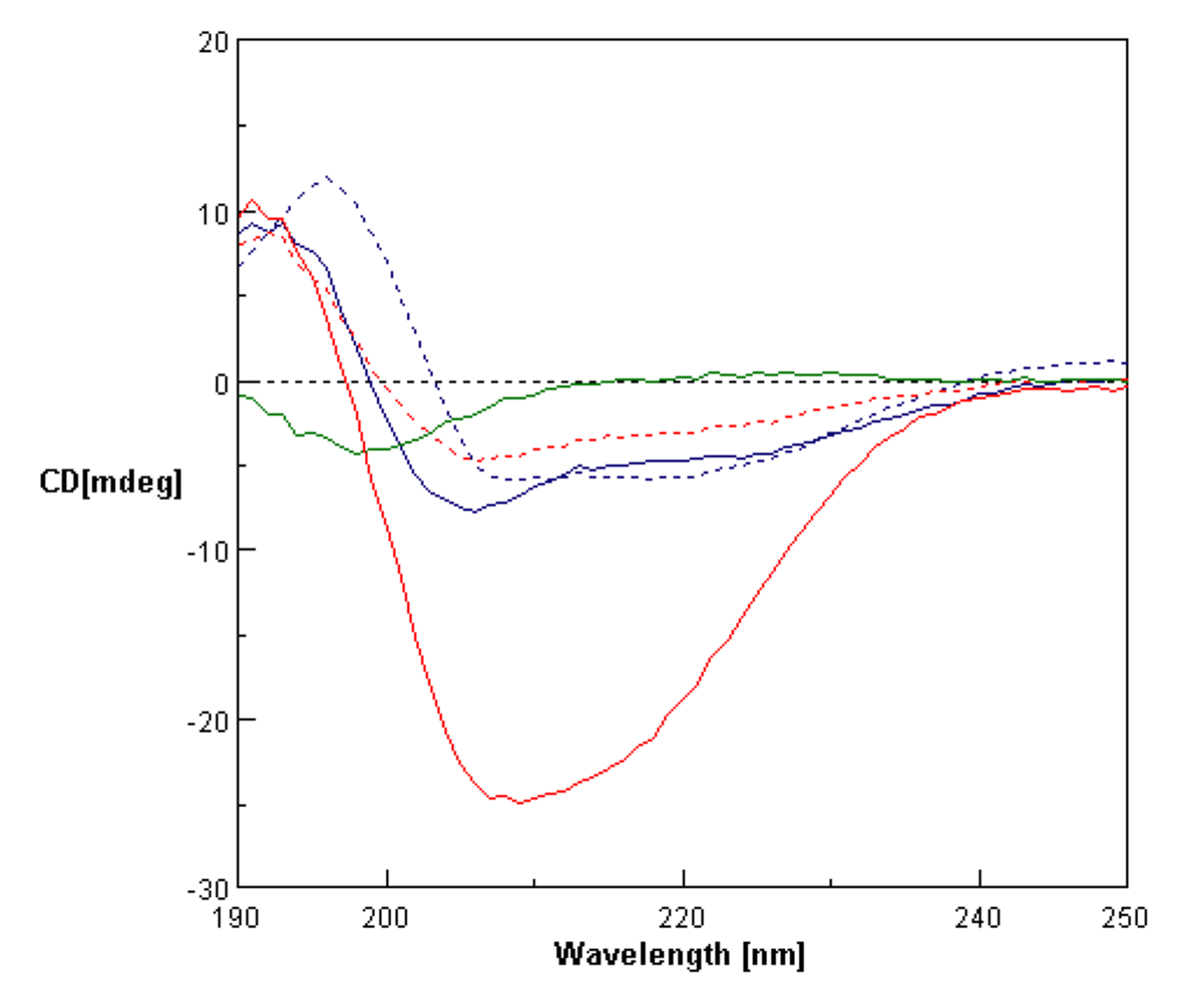

Supplement: Figure S2 — Far-UV CD spectra of the peptides in 2.8 mM SDS under identical conditions. The concentration was 40 μM in all samples. Color code: solid blue, human obestatin (1); dotted blue, non-amidated obestatin (2); solid red, human (6–23)-obestatin (3); dotted red, (11–23)-obestatin (4); and solid green, (16–23)-obestatin (5). (TIF) [file pone.0045434.s002.tif]

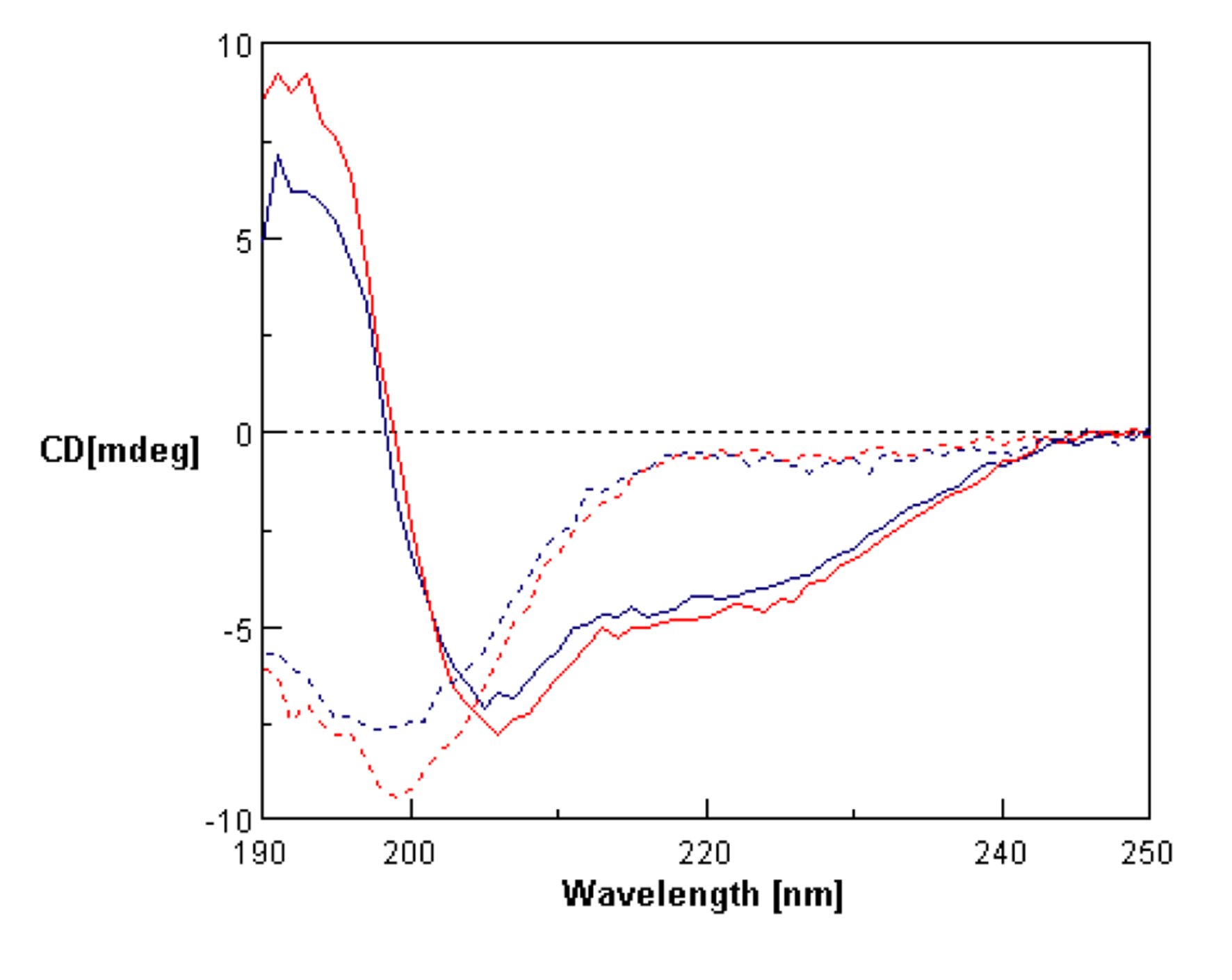

Supplement: Figure S3 — Far-UV CD spectra of human obestatin (1) and mouse obestatin (6) in 25 mM PBS or 2.8 mM SDS under identical conditions. The concentration was 40 μM in all samples. Colour code: solid red, human obestatin (1) in SDS; dotted red, human obestatin (1) in PBS; solid blue, mouse obestatin (6) in SDS; and dotted blue, mouse obestatin (6) in PBS. (TIF) [file pone.0045434.s003.tif]

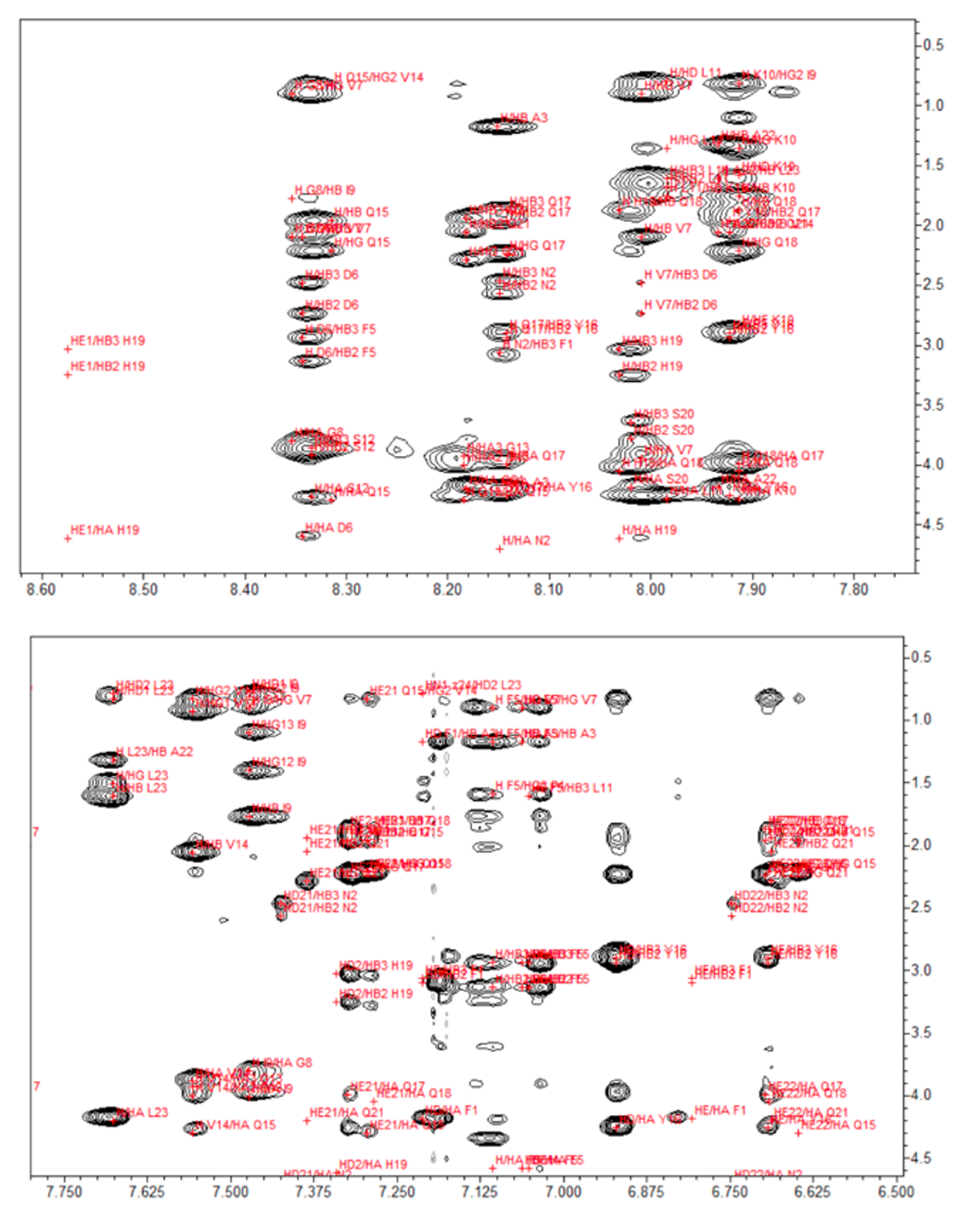

Supplement: Figure S4 — Amide region of 2D NOESY spectra for human obestatin (1) in SDS micelles. (TIF) [file pone.0045434.s004.tif]

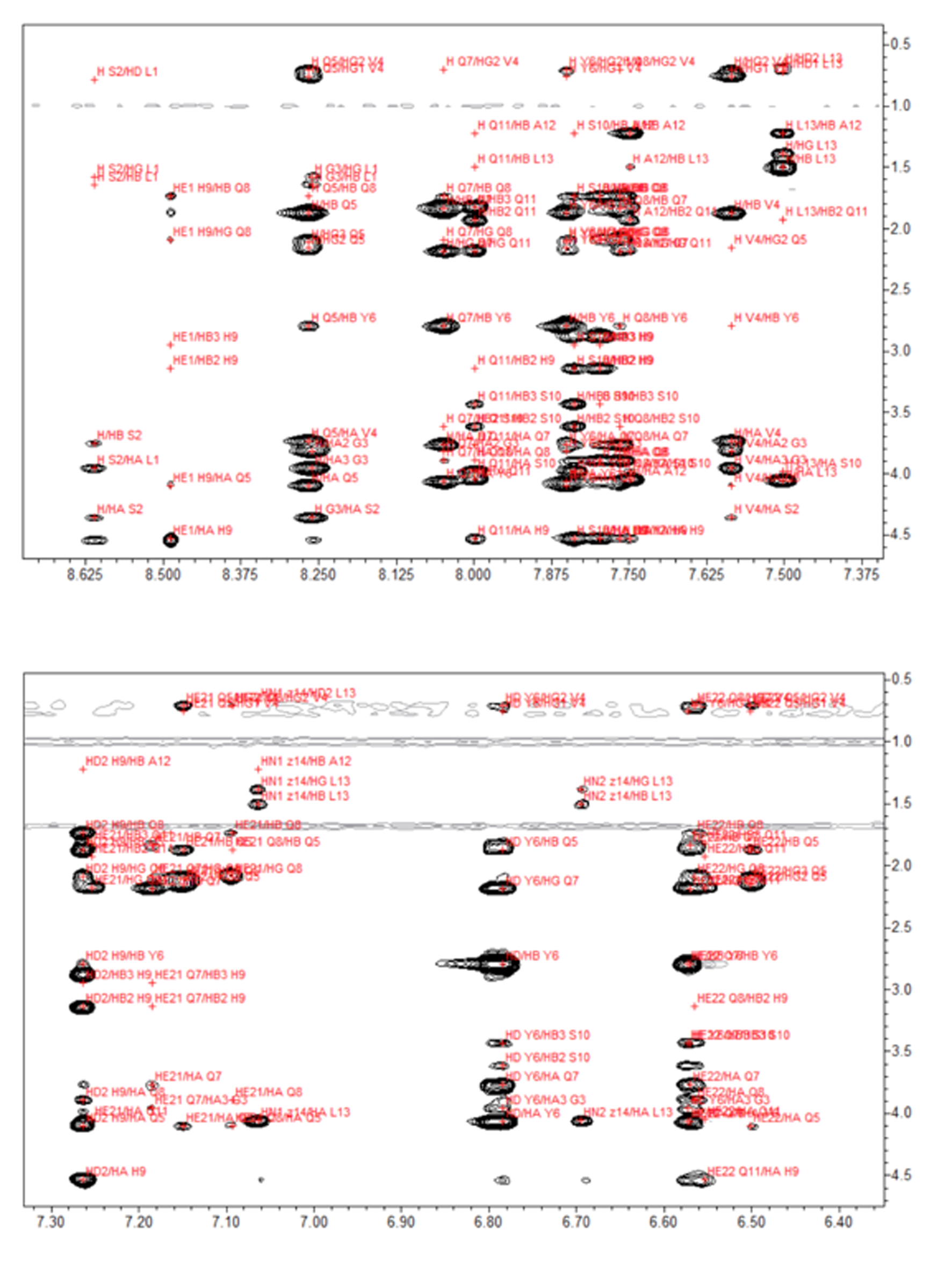

Supplement: Figure S5 — Amide region of 2D NOESY spectra for human (11–23)-obestatin (4) in SDS micelles. (TIF) [file pone.0045434.s005.tif]

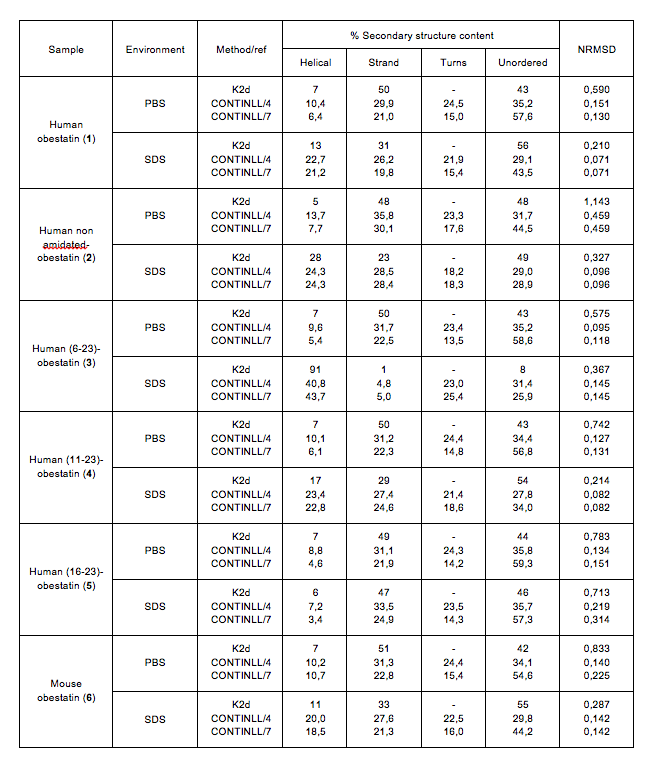

Supplement: Table S1 — Quantification of obestatins secondary structure by CD. The following table describes the quantification of the CD spectra in two different environments. The quantification was performed using the DICHROWEB server and the data were fit using K2d and CONTINLL. The latter was paired with two different data sets for data ranging from 190 to 240 nm. The NRMSD refers to the quality of the curve fitting based on the reference data sets. (TIF) [file pone.0045434.s006.tif]
